# The Banquet Question and the P. M. B. A.

**Published:** 1889-09

**Authors:** A. D. Paulus

**Affiliations:** Flatonia


### THE BANQUET QUESTION—ALSO SOME REMARKS ON A TENDER SUBJECT.

FLATONIA, August 6, 1889.

*Editor Daniel's Texas Medical Journal:*

When, on opening the last number of your always welcome journal, the words, "Requiescat in pace," caught my eye, I was led to believe that another good and true professional brother had crossed the river, and that his brethren would be called upon to contribute the pitiful mite of one dollar for the benefit of his widow, and perhaps children, who had depended on his daily labors for their support. I was much shocked, on reading further, to find that it was not the obituary of a member of the Physicians' Mutual Benefit Association, but of the Association itself. Is it possible? Let me, dear doctor, offer you my condolence, that after a continued struggle through five long years, and in spite of many appeals to your brethren, made so earnestly, you have to surrender your cherished association. What is the matter with the medical profession in Texas, or at least the members of the State Medical Association? Have they become so impoverished as not to be able to contribute so small a sum, and so seldom, too, for the benefit of a brother's widow or orphans? What has become of the so much vaunted charity of the medical profession? Shame, shame, that even some, who have been highly honored by their brothers, should, after paying one or two assessments, default and suffer themselves to be stricken from the roll. Please, dear doctor, publish in one of the numbers of your valuable journal the name of each member, and the number of assessments paid, and then the defunct association "requiescat in pace," even without the hope of resurrection, though by a fine cigar and a long toddy less a day her demise might have been averted.

You ask for the opinion of the members of the State Medical Association as to the advisability of in future doing away with the banquet at the annual meeting. This has been done before; we have met without having the banquet, but the old custom has always been returned to; we have had banquets without wine and with wine. It is, I believe, the time-honored custom with all associations. Since the question has been mooted, I have talked with a number of members in these parts about it. Our opinion is, that it is not our province to prescribe to the members of the profession where we are to meet, how they shall entertain us, but we may, when called upon, express our preferences as well as Drs. Eastland and Ghent, and that preference is the "banquet!" Though we never felt too poor to pay our assessment, most of us are not rich enough to lose the time necessary to attend the annual meeting, and then take a long jaunt to Denver or Pike's Peak. Though our long absence might benefit our patients, it would not benefit us. We do not expect, being plain folks, to be fed on Frenchified delicacies, a plain supper is to us much more palatable, and by that we incur no risk of deranging our stomach, for whose sake a little generous wine (it need not have a manufactured sparkle) is very good. Whoever dces not want it, need not take it, as there is no compulsion in that matter, and whoever cannot partake of it without incurring the risk of getting too much, can let it severely alone. We have no objection to Dr. Ghent's going to Denver, nor Dr. Eastland's sitting on a point of Pike's Peak and viewing the banquet, as Baron Von Humbolt sat on a point of the Cordilleras viewing nature.

With sincere well wishing for the prosperity of the JOURNAL, I am, Doctor, Yours very truly, A. D. PAULUS.

[In October prox., Dr. Paulus will celebrate his fiftieth anniversary as a practitioner of medicine. On the 18th of July he passed his "three-score and ten" and third birthday.—ED.]

